# Pulmonary function in children post -SARS-CoV-2 infection: a systematic review and meta-analysis

**DOI:** 10.1186/s12887-024-04560-1

**Published:** 2024-02-01

**Authors:** Elham Bakhtiari, Nasrin Moazzen

**Affiliations:** 1 Clinical Research Development Unit, Faculty of Medicine, Mashhad University of Medical Sciences, Mashhad, Iran; 2 Allergy Research Center, Mashhad University of Medical Sciences, Mashhad, Iran; 3 Department of Pediatrics, Faculty of Medicine, Mashhad University of Medical Sciences, Mashhad, Iran

**Keywords:** Pulmonary function test, Child, COVID-19, Systematic review, Meta-analysis

## Abstract

**Objective:**

There are some concerns regarding long-term complications of COVID-19 in children. A systematic review and meta-analysis was performed evaluating the respiratory symptoms and pulmonary function, post-SARS-CoV-2 infection.

**Methods:**

A systematic search was performed in databases up to 30 March 2023. Studies evaluating respiratory symptoms and pulmonary function after COVID-19 infection in children were selected. The major outcomes were the frequency of respiratory symptoms and the mean of spirometry parameters. A pooled mean with 95% confidence intervals (CIs) was calculated.

**Results:**

A total of 8 articles with 386 patients were included in meta-analysis. Dyspnea, cough, exercise intolerance, and fatigue were the most common symptoms. The meta-mean of forced expiratory volume (FEV1) and forced vital capacity (FVC) was 101.72%, 95% CI= (98.72, 104.73) and 101.31%, 95% CI= (95.44, 107.18) respectively. The meta-mean of FEV1/FVC and Forced expiratory flow at 25 and 75% was 96.16%, 95% CI= (90.47, 101.85) and 105.05%, 95% CI= (101.74, 108.36) respectively. The meta-mean of diffusing capacity for carbon monoxide was 105.30%, 95%CI= (88.12, 122.49). There was no significant difference in spirometry parameters before and after bronchodilator inhalation.

**Conclusions:**

Despite some clinical respiratory symptoms, meta-results showed no abnormality in pulmonary function in follow-up of children with SARS-CoV-2 infection. Disease severity and asthma background had not confounded this outcome.

**Supplementary Information:**

The online version contains supplementary material available at 10.1186/s12887-024-04560-1.

## Introduction

The coronavirus disease 19 (COVID-19), which rapidly spread worldwide a few years ago, has posed significant challenges to public health, the economy, society, and the environment [1]. This mysterious virus represents very heterogeneous organ involvements. The most prevalent presentations are fever, cough, and anosmia. While early reports primarily indicated mild infections in children, a growing concern has emerged regarding the potential long-term complications of the disease [2]. In early 2020, there were multiple reports of a disease resembling Kawasaki disease in children, characterized by fever, mucocutaneous symptoms, and multi-organ involvement, particularly cardiac issues, often requiring intensive care unit (ICU) admission [3]. Subsequently, additional data suggests higher morbidity and mortality associated with SARS-CoV-2 infection in children.

Autopsy examinations of individuals who have succumbed to COVID-19 have revealed varying degrees of fibroproliferative processes and diffuse alveolar injury, raising concerns about potential respiratory sequelae and persistent impaired pulmonary function in survivors [4]. While most autopsy findings have been in adult patients, the ongoing growth and development of the respiratory system in pediatric patients, especially during infancy and early childhood, may render them more susceptible to pulmonary complications [5].

Expected pathophysiology indicates a higher likelihood of a restrictive pattern. Available data indicates that abnormal diffusion capacity for carbon monoxide (DLCO), which correlates with the severity of acute illness, is a common result in pulmonary function tests (PFT) of post-acute patients. Ground glass opacities are frequently observed in high-resolution CT scans [6, 7].

A recent meta-analysis has revealed that 77% of infected patients with SARS-Cov2 exhibited abnormal lung CT findings during the acute phase [2]. Furthermore, several studies have documented persistent post-COVID-19 respiratory symptoms, with 25–42% of patients reporting moderate to severe dyspnea 4–8 weeks after hospital discharge [8].

Pulmonary function testing is a valuable method for assessing long-term pulmonary complications in survivors of COVID-19, providing safe, objective, and accurate measures of airway restriction and obstruction [5]. Notably, despite the significant impact on pediatric patients, there is currently a scarcity of systematic reviews and meta-analyses regarding the long-term respiratory outcomes in this population post-SARS-CoV-2 infection. This review aims to consolidate available evidence and identify research gaps to guide future investigations into the long-term effects of COVID-19 on pediatric respiratory health.

## Methods

### Literature search strategy and study selection

Relevant databases including Medline, Web of Sciences, Embase, and Scopus were searched comprehensively to assess literature up to 30 March 2023 in the English language. The search terms included “COVID-19 or coronavirus 2019” or “SARS-CoV-2” AND (“pulmonary function” OR “pulmonary diseases” OR “lung problem” OR “lung Sequelae) AND “children” or “pediatrics”. They were used separately or/or in combinations to obtain the eligible documents. The references of eligible articles were searched manually to find additional relevant papers. This study was conducted in adherence to the Preferred Reporting Items for Systematic Reviews and Meta-Analysis (PRISMA) statement [9]. The current study was based on published articles. Therefore, the consent form was not needed. Two researchers (EB and NM) independently reviewed the titles and abstracts of all studies to identify relevant articles. Articles were included according to the following criteria: [1] English language, longitudinal or cross-sectional studies evaluating the pulmonary function and clinical symptoms of children after COVID-19 infection [2] spirometry parameters have been measured [3] the study population were pediatrics. Case reports, case series, letters to editors, unpublished reports, duplications and laboratory studies were excluded. In duplicate articles, the recent and more informative one was included. Articles were assessed using the Newcastle-Ottawa Quality Assessment Scale (NOS) for cohort and cross-sectional studies [10]. NOS has included selection, comparability, and outcome sections. The score range is varying between 0 and 10. A study obtained 5–6 stars was considered satisfactory (fair) quality. Studies with 7–8 stars were considered as good and studies with 9–10 stars were considered as very good quality. Studies with satisfactory (fair) quality and higher were included in the study.

### Data extraction

Two independent researchers (EB and NM) extracted data from eligible studies. A data collection sheet was used for data extraction. Disagreement was judged by consensus or by a third party. Data from each study included the author’s name, year of publication, county of study, study design, number of studied patients, the age range of children, the interval between COVID-19 infection or infection recovery (whichever is mentioned), and pulmonary function assessment, mean ± standard deviation (SD) of spirometry parameters and frequency of respiratory symptoms.

### Statistical analysis

The meta-mean with a 95% confidence interval (CI) was calculated based on the mean and SD of spirometry parameters. If a study only reported the median, range, and/or inter-quartile range (IQR); mean and SD were estimated, according to Hozo et al. [11]. The Cochran Q statistic and inconsistency index (I^2^) were used to assess the heterogeneity among studies. If I^2^ was more than 50%, and the p-value was lesser than 0.05, heterogeneity was considered significant. The random effect model was used for significant heterogeneity, whereas the fixed effect model was applied for non-significant heterogeneity. To assess the stability of the results, the sequential omitting of individual studies in the meta-analysis was performed using sensitivity analysis. Subgroups were analyzed based on disease severity. Probable confounders were verified using meta-regression. The standardized mean difference (SMD) was calculated in studies, which measured spirometry parameters twice. Publication bias was assessed using Egger’s linear regression test. Agreement between authors in data selection and extraction was assessed using Cohen’s kappa statistic. Statistical analysis was performed using the Comprehensive Meta-Analysis (CMA) computer program (Biostat, Englewood, NJ). A p-value less than 0.05 was considered statistically significant.

## Results

### Literature search

**Fig. 1:**
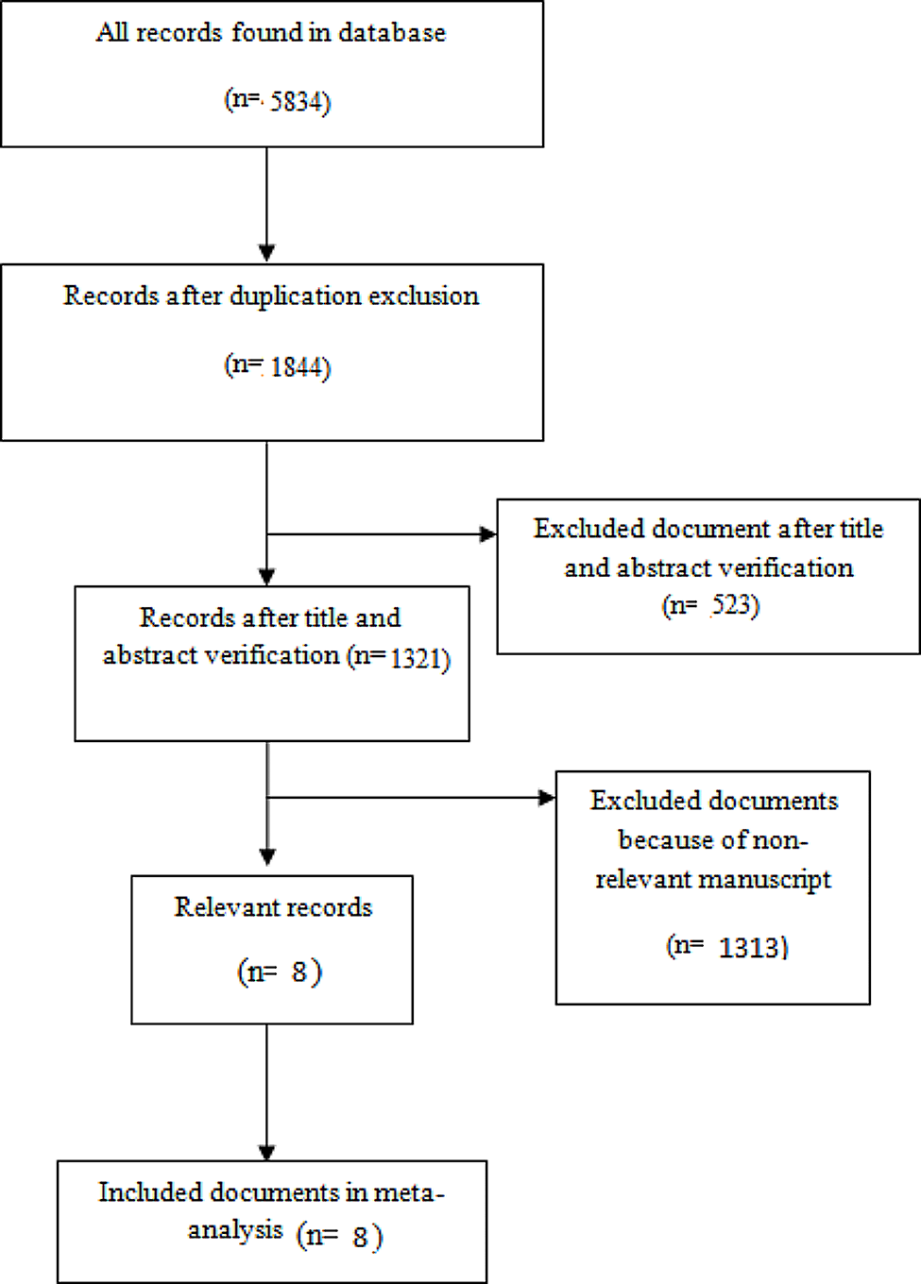
Diagram of study selection

A diagram of study selection is presented in Fig. 1. In a primary search, 5834 papers were obtained evaluating the respiratory function and symptoms after COVID-19 in children. During the screening process, some studies were excluded because they were review articles, case series, or conference abstracts. Some studies were excluded because they studied adults, or evaluated respiratory function and symptoms during COVID-19 infection. Some studies were excluded because of duplication. Finally, eight articles including 386 patients were enrolled in the present review [12–18]. Eligible studies were including 6 cross sectionals [12, 13, 15, 16, 18, 19] and 2 longitudinal studies [14, 17]. In two studies the z score of spirometry parameters were reported [15, 18]. Details of eligible studies are presented in Table 1. Cohen’s kappa statistic for interrater agreement in data selection and extraction was 0.98, p p-value < 0.0001.

**Table 1:** Characteristics of eligible studies included in meta-analysis

Name	Study design	Sample size	Time from infection/recovery to PFT (month)	Disease severity	Asthma history	FEV1 (%)	FVC (%)	FEV1/FVC	FEF25-75 (%)	DLCO (%)
Palacios (USA)	Cohort	82	6.7 (4.9, 8.6)	All types	Not Excluded	104 (97–111)	104 ± 13	NR	96 (102–126)	118 ± 17
Ipek (Turkey)	Cross sectional	34	49 (36)*	All types	Excluded	98.67 ± 14.93	94.21 ± 13.68	101.06 ± 24.89	101.85 ± 24.89	NR
Ozturk (Turkey)	Cross sectional	50	3	All types	Excluded	105.18 ± 15.92	110.38 ± 12.33	94.18 ± 8.76	95.82 ± 26.31	89.65 ± 8.76
Chiara (Italy)	Observational	61	10 ± 4	Asymptomatic	Excluded	98.38 (94.38–104.39)	93.35 (89.56–103.56)	105.34 (100.06–107.46)	105.26 (92.35–117.33)	NR
Bottino (Italy)	Cross sectional	16	67 (49–91)*	Asymptomatic	Excluded	96 (94–102)	95 (87–100)	92 (87–97)	NR	119 (111–132)
Dobkin (USA)	Cohort	29	6 weeks or more	All types	Not Excluded	107 ± 12	110 ± 16	86 ± 8	100 ± 23	95 ± 17
Knoke (Germany)^+^	Cross sectional	73	2.59 (0.4, 6)	All types	Not Excluded	0.3 ± 1.04	−0.21 ± 1.06	-	-	2.03 ± 2.35
Boguslawski (Poland) ^+^	Observational	41	Up to 6 months	All types	Not Excluded	−0.76 (− 1.36,2.01)	0.49 (− 0.5,1.7)	0.04 (− 1.05, 0.64)	-	0.18 (− 0.84,0.61)

PFT: pulmonary function test, DLCO: diffusing capacity for carbon monoxide, FVC: forced vital capacity, FEV1: forced expiratory volume, FEF25-75%: Forced expiratory flow at 25 and 75% of the pulmonary volume, NR: not reported, * day # quantitative data were presented as mean ± standard deviation or median (inter quartile range or range), + parameters were reported as Z score

### Systematic review of respiratory symptoms and spirometry parameters

Eight studies from Italy, Turkey, Germany, Poland, and the USA evaluated the clinical symptoms and respiratory functions in children post-COVID-19 infection. The sample size ranged from 16 to 82 participants. Totally 386 patients were studied. The age of patients was varying from 5 to 18 years. Among 386 patients, 204 were females (52.84%). Respiratory function was evaluated at least 6 weeks after infection. Cough, dyspnea, exercise intolerance, fatigue and chest pain were common clinical symptoms. In two studies no respiratory symptoms were reported [13, 19]. Data are presented in Table 2. Regarding spirometry parameters, four studies reported that COVID-19 did not affect respiratory function [12–15] and four studies reported that it could affect pulmonary function [16–19]. In four studies patients with a history of asthma were excluded [12, 13, 16, 19]. In two studies spirometry parameters were measured before and after bronchodilator inhalation [12, 17]. In six studies all types of COVID-19 were included [14, 16–19], and in two studies only mild or asymptomatic patients were enrolled [12, 13]. Among 8 eligible studies, two studies reported the z score of spirometry parameters [15, 18]. So they were analyzed separately.

**Table 2:** Frequency of clinical respiratory symptoms at the time of follow-up

Study name	Clinical respiratory symptoms after infection
Bogusławski (2023)	Persistent symptoms were presented in 17.1% of children. They included: Decreased exercise tolerance (57.1%) Dyspnea (42.9%) Cough (42.9%) Fatigue (28.6%) Sleeping difficulties (14.3%) Impaired concentration (14.3%) Lack of appetite (14.3%)
Chiara (2022)	No symptom at rest No exercise-induced respiratory symptoms
Ipek (2022)	Not presented in the article
Knoke (2022)	Any long-term complaints were reported in 27.1% of patients. They included: Fatigue (14.28%) Loss of smell/taste (10%) Breathing problems (8.57%) Headache (4.28%) Cough (2.85%)
Ozturk (2022)	Respiratory symptoms were reported in 28% of patients. They included: Dyspnea (35.7%) Exertional dyspnea (35.7%) Cough (21.4%) Chest pain and tightness (21.4%)
Palacios (2022)	Respiratory symptoms were reported in 48.7% of patients. They included: Shortness of breath during exercise (67.5%) Chest pain (20%) Dyspnea (15%) Cough (12.5%)
Bottino (2021)	All patients were free of respiratory symptoms at the time of follow-up
Dobkin (2021)	Persistent dyspnea and/or exertional dyspnea (96.6%) Cough (51.7%) Exercise intolerance (48.3%) Fatigue (13.8%) One subject had an ongoing supplemental oxygen requirement

### Meta-analysis of clinical symptoms and pulmonary function

Clinical symptoms were presented in five articles. Cough was reported in five studies [14–18]. The meta-proportion for cough was 0.25, 95% CI= (0.06, 0.44), I^2^ = 92.38. Dyspnea was reported in five studies [14–18]. The meta-proportion for dyspnea was 0.4, 95% CI= (-0.03, 0.83), I^2^ = 98.94. Fatigue was reported in four studies [12, 14, 15, 18]. The meta-proportion for fatigue was 0.16, 95% CI= (0.11, 0.21), I^2^ = 0. Dyspnea in exercise or exercise intolerance was reported in three studies [16–18]. The meta-proportion for dyspnea in exercise or exercise intolerance was 0.55, 95% CI= (0.40, 0.71), I^2^ = 57.74. Chest pain was reported in two studies [16, 17]. The meta-proportion for chest pain was 0.20, 95% CI= (0.09, 0.31), I^2^ = 0. Other non-respiratory clinical symptoms included loss of appetite [18], impaired concentration [18], sleeping difficulties [18], headache [15], and loss of smell/taste [15] each one was presented in in the one study.

**Fig. 2:**
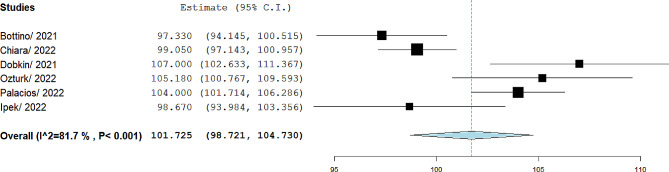
Pooled mean of FEV1 in included studies

The spirometry parameters were reported in eight articles [12–19]. In Knoke and Bogusławski studies the z score of parameters was reported [15, 18]. In two studies, spirometry parameters were reported pre- and post-bronchodilator inhalation [12, 17]. In five studies, spirometry parameters were reported without bronchodilator inhalation [13–16, 19]. The FEV1, FVC, and FEV1/FVC were reported in 345 patients. According to random effect modeling in the meta-analysis, the mean of FEV1 was 101.72%, 95% CI= (98.72, 104.73), I^2^ = 81.7. The forest plot is shown in Fig. 2.

**Fig. 3:**
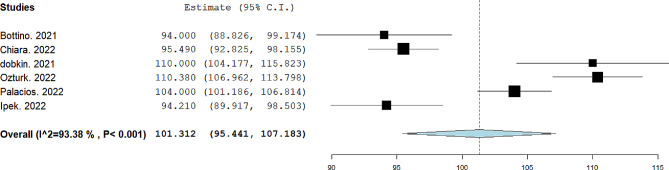
Pooled mean of FVC in included studies

**Fig. 4:**
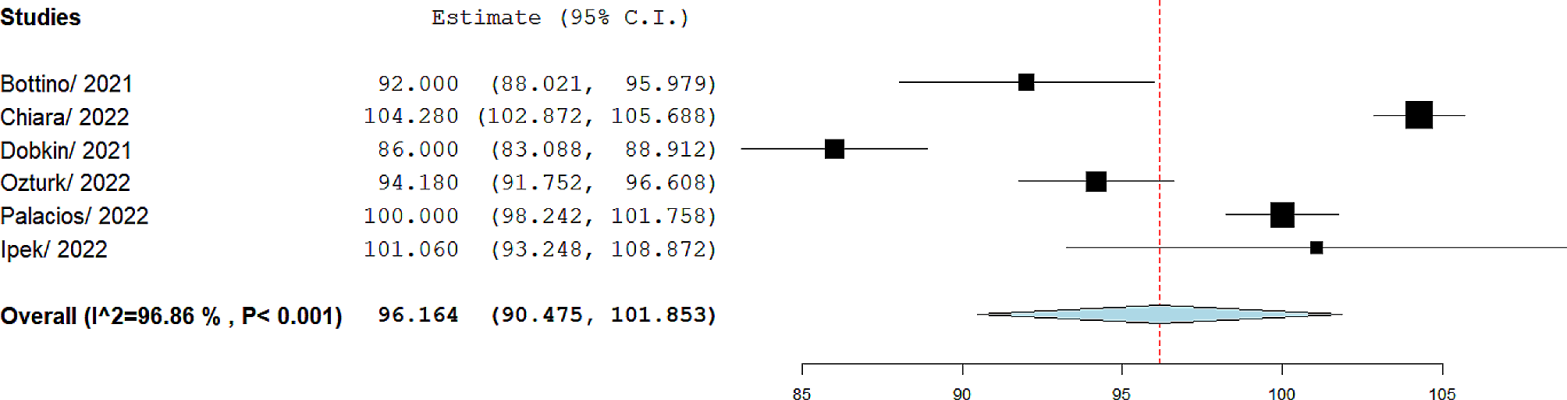
Pooled mean of FEV1/FVC in included studies

**Fig. 5:**
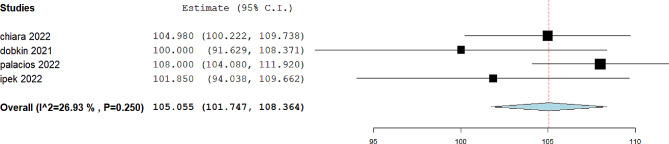
Pooled mean of FEF25-75 in included studies

**Fig. 6:**
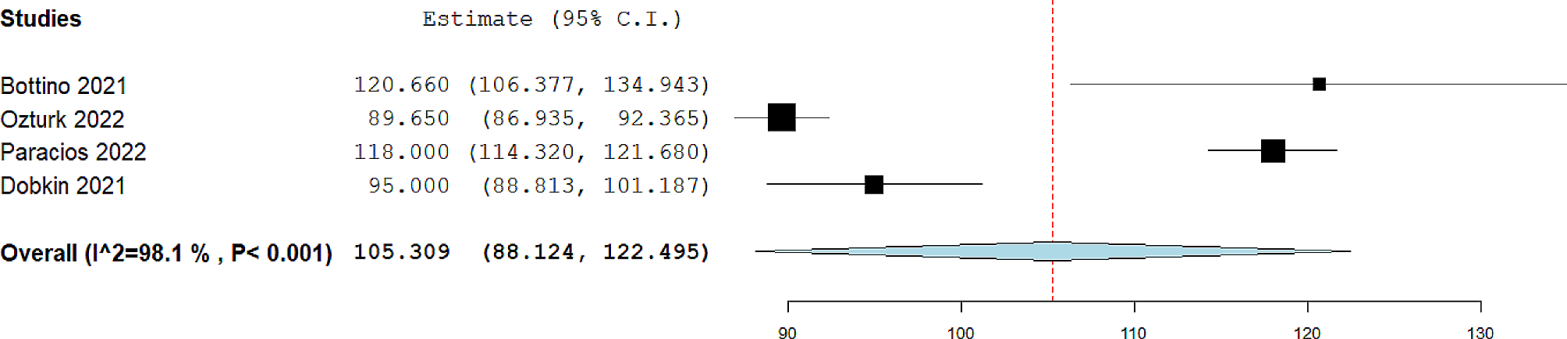
Pooled mean of DLCO in included studies

The pooled mean of FVC was 101.31%, 95% CI= (95.44, 107.18), I^2^ = 93.38. The forest plot is shown in Fig. 3. The pooled mean of FEV1/FVC was 96.16%, 95% CI= (90.47, 101.85), I^2^ = 96.86. The forest plot is shown in Fig. 4. The pooled mean of FEF25-75 was reported in four studies [13, 14, 17, 19]. The pooled mean of FEF25-75 was 105.05%, 95% CI= (101.74, 108.36), I^2^ = 26.93. The forest plot is shown in Fig. 5. The pooled mean of total lung capacity (TLC) was reported in two studies. The pooled mean of TLC was 99.52%, 95% CI= (84.1, 114.94), I^2^ = 90.88. The mean of DLCO was reported in four studies [12, 14, 16, 17]. The pooled mean of DLCO was 105.30%, 95% CI= (88.12, 122.49), I^2^ = 98.10. The forest plot is shown in Fig. 6. The pooled mean of lung clearance index (LCI) was 7.31, 95% CI= (6.49, 8.2), I^2^ = 90.88. In two studies, FEV1, FVC, and FEV1/FVC were reported before and after bronchodilator inhalation [12, 17]. Meta-analysis confirmed that there was no significant difference in spirometry parameters before and after bronchodilator inhalation. The SMD for FEV1 was − 0.21, 95% CI= (-0.65, 0.23), p value = 0.35, I^2^ = 38.3. The SMD for FVC1 was − 0.07, 95% CI= (-0.35, 0.21), p value = 0.14, I^2^ = zero. The SMD for FEV1/FVC was − 0.29, 95% CI= (-0.58, 0.01), p value = 0.07, I^2^ = zero.

### Meta-analysis of spirometry parameters according to Z score

According to meta-analysis, the mean Z score of FEV1 and FVC was 0.28, 95% CI= (-0.02, 0.59), I^2^ = 0 and 0.08, 95% CI= (-0.65, 0.81), I^2^ = 69.90 respectively. The meta-mean Z score of DLCO was 1.14, 95% CI= (-1.12, 3.40), I^2^ = 97.25.

### Heterogeneity analysis

Subgroup analysis according to the severity of the disease and sensitivity analysis were carried out evaluating the possible source of heterogeneity. In two studies [12, 13] patients with asymptomatic COVID-19 were studied and in four studies all types of disease (asymptomatic and symptomatic) were studied [14, 16, 17, 19]. According to meta-analysis the pooled mean of FEV1 in the asymptomatic subgroup was 98.59%, 95%CI= (96.96, 100.23), I^2^ = zero. The pooled mean of FEV1 in symptomatic subgroup was 103.91%, 95%CI= (101.08, 106.74), I^2^ = 53.27. The pooled mean of FVC in asymptomatic subgroup was 95.17%, 95%CI= (92.80, 97.54), I^2^ = zero. The pooled mean of FVC in the symptomatic subgroup was 104.62%, 95%CI= (98.00, 111.24), I^2^ = 91.17. The pooled mean of FEV1/FVC in the asymptomatic subgroup was 98.28, 95%CI= (86.25, 110.31), I^2^ = zero. The pooled mean of FEV1/FVC in the symptomatic subgroup was 94.99%, CI= (88.19, 101.79), I^2^ = 95.65.

Meta-regression showed neither disease severity nor asthma comorbidity had a significant effect on the pooled mean of FEV1 (p value = 0.35 and 0.21 respectively) and FVC (p value = 0.80 and 0.51 respectively). In sensitivity analysis, the effect of each study on the pooled mean was assessed. There was no major deviation from the pooled mean by omitting studies in FEV1, FVC FEV1/FVC, FEF25-75, and DLCO outcomes indicating the stability and robustness of the results (Data not shown).

### Publication bias

Egger’s regression asymmetry test was used to explore the probable publication bias for FEV1, FVC and DLCO parameters. The Egger’s test result provided no significant bias across the included studies (p value = 0.39, 0.69 and 0.53 respectively).

## Discussion

After the widespread distribution of COVID-19 in pediatric patients, one of the most important issues was long-lasting complications in the next generation. Current evidence has mentioned an increased risk of diabetes mellitus type I and severe diabetes ketoacidosis in children infected by SARS-CoV-2 [20]. Autoimmune disorders might be more expected in coming years due to the impact of COVID-19 on the immune system [21]. One of the most prevalent symptoms in children infected by SARS-CoV-2 is respiratory manifestation [22]. There are increasing evidences of the pulmonary sequel, especially in the adult population after infection [23]. To the best of our knowledge present study is the first systematic review and meta-analysis evaluating the impact of COVID-19 on the respiratory system of the younger generation in the long-term.

Infection generally was mild and most of the patients had no or only mild symptoms during infection. Among the included studies, at least 6 articles enumerated respiratory manifestations in the follow-up of pediatric population with a history of SARS-Cov 2 infection. Dyspnea (in rest and/or in the exercise) and cough are two of the most prevalent symptoms. Fatigue and chest pain are other symptoms. However, none of the studies graded these complaints for severity. It seems important to know about the severity of these manifestations because all spirometry parameters were in the normal range. Two studies evaluated post-bronchodilator parameters. Their results showed no reversible obstructive changes in the airways of children with a history of COVID-19. Fortunately, all studies reported FEF 25–75%. It is one of the most sensitive measures of obstructive diseases in peripheral airways [24–27]. The meta-mean of FEF25-75% was 105.05%, which was in the normal range. According to our meta-results, no obstructive disease in the studied population was detected.

One of the most expected involved areas in the respiratory system during COVID-19 is alveolar epithelial cells [28, 29]. It seems that peripheral airways with an internal diameter of less than 2 millimeters are more prone to impairment after SARS-CoV-2 infection. While these parts of the respiratory system represent 90% of total lung capacity but only have a role in less than 20% of airflow [30, 31]. So, simple spirometry which measures FEV1 and FVC hardly detects the early stages of pulmonary involvement after COVID-19. Measuring diffusion capacity is more sensitive in detection of pulmonary diseases, especially in the early stages [32]. Unfortunately, only 177 out of 238 participants had DLCO values. However, according to meta-results the mean of DLCO was within normal range (108.97%, 95%CI: (86.15, 131.79)). Another parameter that can reveal the early stages of peripheral airway pathology and impaired PFT, is LCI which is measured by multiple breath wash (MBW). In our eligible studies, two studies have reported this index. However, its Meta mean was within the normal range (7.31). Two studies reported TLC. The Meta mean was 95.52% which is in the normal range. A meta-analysis in adults was evaluated pulmonary function post-COVID 19 infection. Results showed decreased DLCO in nearly 40% of survivors [33, 34]. Decreased DLCO might be an early indicator of interstitial lung diseases even before a change in lung volumes [35, 36]. Chronic interstitial pneumonia and diffuse alveolar hemorrhage are demonstrated in a few studies, which have reported histological findings in autopsy [37–39]. Patients with SARS-CoV-2 may have pulmonary fibrosis, which is considered a defined sequel of barotrauma. All of these pathologies can impair carbon monoxide diffusion capacity [40]. In the present study, one explanation for normal DLCO may be a none-severe infection in most of the studied children. We have tried to evaluate the impact of disease severity on spirometry parameters. However, there were not significant differences between the results in symptomatic and asymptomatic patients. Future studies with longer periods of follow-up and evaluating patients with more severe respiratory presentation are needed. In addition, severity grading of long-lasting symptoms should be considered. So evaluation and analysis of pulmonary sequel will be much accurate. We had also heterogeneity in the atopy and asthma background of our included studies. However, according to meta-regression chronic pulmonary disease (such as asthma) had not a significant effect on the pooled mean of major outcomes.

Less severity of respiratory system involvement in children infected by SARS-CoV-2 compared with adults, might be a possible explanation for different outcomes between them [3]. In addition, preexisting diseases in adults like chronic respiratory diseases, cardiac diseases, and diabetes mellitus may induce impairment in pulmonary function. On the other hand, children during infancy and preschool age usually have a more severe course during infection [41, 42]. Because the majority of our included participants were teenagers, more studies, which can evaluate pulmonary sequel in infants and young toddlers, should be designed. In addition, different variants of SARS-CoV-2 like Delta or Omicron had resulted to different presentation and probably different outcomes. Therefore, studies, which determine the type of variants, may be useful. It is possible that the pulmonary sequel of survived children is so tiny that routine pulmonary function tests cannot detect abnormalities. It is useful to design exercise-challenging studies in survived children after COVID-19 to detect subtle or mild changes in pulmonary function.

## Conclusion

Although more evidence is needed, our review showed no abnormality in the pulmonary function test despite the existence of some clinical respiratory symptoms in the follow-up of children with a SARS-CoV-2 infection history. Disease severity and asthma background had not confounded this outcome.

### Limitation

There are some limitations regarding the present study: (A) despite an attempt for a comprehensive search, it may be that some eligible articles were missed. (B) Eligible studies were observational and they were threatened with bias at different levels. It may affect the meta-results.

### Electronic supplementary material

Below is the link to the electronic supplementary material.

Supplementary Material 1

## Data Availability

The data that support the findings of this study are openly available at the web space (as original articles).

## References

[1] Chakraborty I, Maity P (2020). COVID-19 outbreak: Migration, effects on society, global environment and prevention. Sci Total Environ.

[2] Mair M, Singhavi H, Pai A, Singhavi J, Gandhi P, Conboy P (2021). A meta-analysis of 67 studies with presenting symptoms and laboratory tests of COVID‐19 patients. Laryngoscope.

[3] Jurado Hernandez JL, Alvarez Orozco IF (2021). COVID-19 in children: respiratory involvement and some differences with the adults. Front Pead.

[4] Maiese A, Manetti AC, La Russa R, Di Paolo M, Turillazzi E, Frati P (2021). Autopsy findings in COVID-19-related deaths: a literature review. Forensic Sci Med Pathol.

[5] Burks AW, Bacharier LB, Hershey GKK, Peebles RS, O’Hehir RE, Broide DH, et al. Middleton’s allergy E-Book: principles and practice. Elsevier Health Sciences; 2019.

[6] Adeloye D, Elneima O, Daines L, Poinasamy K, Quint JK, Walker S (2021). The long-term sequelae of COVID-19: an international consensus on research priorities for patients with pre-existing and new-onset airways disease. The Lancet Respiratory Medicine.

[7] Huang C, Huang L, Wang Y, Li X, Ren L, Gu X (2021). 6-month consequences of COVID-19 in patients discharged from hospital: a cohort study. The Lancet.

[8] Huang L, Li X, Gu X, Zhang H, Ren L, Guo L, et al. Health outcomes in people 2 years after surviving hospitalisation with COVID-19: a longitudinal cohort study. The Lancet Respiratory Medicine; 2022. 10.1016/S2213-2600(22)00126-6 PMC9094732 35568052

[9] Moher D, Shamseer L, Clarke M, Ghersi D, Liberati A, Petticrew M (2015). Preferred reporting items for systematic review and meta-analysis protocols (PRISMA-P) 2015 statement. Syst Reviews.

[10] Margulis AV, Pladevall M, Riera-Guardia N, Varas-Lorenzo C, Hazell L, Berkman ND (2014). Quality assessment of observational studies in a drug-safety systematic review, comparison of two tools: the Newcastle–Ottawa scale and the RTI item bank. Clin Epidemiol.

[11] Hozo SP, Djulbegovic B, Hozo I (2005). Estimating the mean and variance from the median, range, and the size of a sample. BMC Med Res Methodol.

[12] Bottino I, Patria MF, Milani GP, Agostoni C, Marchisio P, Lelii M (2021). Can asymptomatic or non-severe SARS-CoV-2 infection cause medium-term pulmonary sequelae in children?. Front Pead.

[13] Di Chiara C, Carraro S, Zanconato S, Cozzani S, Baraldi E, Giaquinto C (2022). Preliminary evidence on pulmonary function after asymptomatic and mild COVID-19 in children. Children.

[14] Leftin Dobkin SC, Collaco JM, McGrath-Morrow SA (2021). Protracted respiratory findings in children post‐SARS‐CoV‐2 infection. Pediatr Pulmonol.

[15] Knoke L, Schlegtendal A, Maier C, Eitner L, Lücke T, Brinkmann F. Pulmonary function and long-term respiratory symptoms in children and adolescents after COVID-19. Front Pead. 2022;10. 10.3389/fped.2022.851008 PMC9081758 35547532

[16] Öztürk GK, Beken B, Doğan S, Akar HH. Pulmonary function tests in the follow-up of children with COVID-19. Eur J Pediatrics. 2022:1–9. 10.1007/s00431-022-04493-w PMC9072762 35522314

[17] Palacios S, Krivchenia K, Eisner M, Young B, Ramilo O, Mejias A et al. Long-term pulmonary sequelae in adolescents post‐SARS‐CoV‐2 infection. Pediatr Pulmonol. 2022. 10.1002/ppul.26059 PMC9349789 35775163

[18] Bogusławski S, Strzelak A, Gajko K, Peradzyńska J, Popielska J, Marczyńska M (2023). The outcomes of COVID-19 pneumonia in children—clinical, radiographic, and pulmonary function assessment. Pediatr Pulmonol.

[19] Ipek S, Gungor S, Gullu UU, Kizildag B, Ozkars MY, Yurttutan S et al. Evaluation of Pulmonary functions after Discharge in Pediatric patients with COVID-19: a prospective study. The Medical Bulletin of Sisli Etfal Hospital. 10.14744/SEMB.2022.36047 PMC9580971 36304229

[20] Rahmati M, Keshvari M, Mirnasuri S, Yon DK, Lee SW, Il Shin J et al. The global impact of COVID-19 pandemic on the incidence of pediatric new‐onset type 1 diabetes and ketoacidosis: a systematic review and meta‐analysis. J Med Virol. 2022. 10.1002/jmv.27996 PMC9350204 35831242

[21] Anaya J-M, Herrán M, Beltrán S, Rojas M. Is post-COVID syndrome an autoimmune disease? Expert Rev Clin Immunol. 2022(just-accepted). 10.1080/1744666X.2022.2085561 35658801

[22] Mansourian M, Ghandi Y, Habibi D, Mehrabi S (2021). COVID-19 infection in children: a systematic review and meta-analysis of clinical features and laboratory findings. Archives de Pédiatrie.

[23] Boutou AK, Georgopoulou A, Pitsiou G, Stanopoulos I, Kontakiotis T, Kioumis I (2021). Changes in the respiratory function of COVID-19 survivors during follow‐up: a novel respiratory disorder on the rise?. Int J Clin Pract.

[24] Ciprandi G, Cirillo I, Klersy C, Marseglia GL, Vizzaccaro A, Pallestrini E (2006). Role of FEF25–75 as an early marker of bronchial impairment in patients with seasonal allergic rhinitis. Am J Rhinol.

[25] Patterson GM, Wilson S, Whang JL, Harvey J, Agacki K, Patel H (1996). Physiologic definitions of obliterative bronchiolitis in heart-lung and double lung transplantation: a comparison of the forced expiratory flow between 25% and 75% of the forced vital capacity and forced expiratory volume in one second. J Heart lung Transplantation: Official Publication Int Soc Heart Transplantation.

[26] Malerba M, Radaeli A, Olivini A, Damiani G, Ragnoli B, Sorbello V (2016). Association of FEF25-75% impairment with bronchial hyperresponsiveness and airway inflammation in subjects with asthma-like symptoms. Respiration.

[27] Bird Y, Staines-Orozco H (2016). Pulmonary effects of active smoking and secondhand smoke exposure among adolescent students in Juárez. Mexico Int J Chronic Obstr Pulmonary Disease.

[28] Li X, Ma X (2020). Acute respiratory failure in COVID-19: is it typical. ARDS? Crit care.

[29] Moazzen N, Imani B, Aelami MH, Haghi NSM, Kianifar HR, Khoushkhui M (2020). How to boost our immune system against coronavirus infection?. Archives of Bone and Joint Surgery.

[30] Hildebrandt J, Rahn A, Kessler A, Speth F, Fischer D-C, Ballmann M (2021). Lung clearance index and diffusion capacity for CO to detect early functional pulmonary impairment in children with rheumatic diseases. Pediatr Rheumatol.

[31] Macklem PT (1998). The physiology of small airways. Am J Respir Crit Care Med.

[32] Macintyre N, Crapo R, Viegi G, Johnson D, Van der Grinten C, Brusasco V (2005). Standardisation of the single-breath determination of carbon monoxide uptake in the lung. Eur Respir J.

[33] Torres-Castro R, Vasconcello-Castillo L, Alsina-Restoy X, Solís-Navarro L, Burgos F, Puppo H (2021). Respiratory function in patients post-infection by COVID-19: a systematic review and meta-analysis. Pulmonology.

[34] Mo X, Jian W, Su Z, Chen M, Peng H, Peng P et al. Abnormal pulmonary function in COVID-19 patients at time of hospital discharge. Eur Respir J. 2020;55(6). 10.1183/13993003.01217-2020 PMC7236826 32381497

[35] Oliveira R, Ribeiro R, Melo L, Grima B, Oliveira S, Alves J. Connective tissue disease-associated interstitial lung disease. Pulmonology. 2020. 10.1016/j.pulmoe.2020.01.004 32044296

[36] Krauss E, El-Guelai M, Pons-Kuehnemann J, Dartsch RC, Tello S, Korfei M (2020). Clinical and functional characteristics of patients with unclassifiable interstitial lung disease (uILD): long-term follow-up data from European IPF Registry (eurIPFreg). J Clin Med.

[37] Zhang H, Zhou P, Wei Y, Yue H, Wang Y, Hu M (2020). Histopathologic changes and SARS-CoV-2 immunostaining in the lung of a patient with COVID-19. Ann Intern Med.

[38] Pernazza A, Mancini M, Rullo E, Bassi M, De Giacomo T, Rocca CD (2020). Early histologic findings of pulmonary SARS-CoV-2 infection detected in a surgical specimen. Virchows Arch.

[39] Calabrese F, Pezzuto F, Fortarezza F, Hofman P, Kern I, Panizo A (2020). Pulmonary pathology and COVID-19: lessons from autopsy. The experience of European Pulmonary pathologists. Virchows Arch.

[40] Chippa V, Aleem A, Anjum F. Post acute coronavirus (COVID-19) syndrome. 2021. 34033370

[41] Taheri L, Gheiasi SF, Taher M, Basirinezhad MH, Shaikh ZA, Dehghan Nayeri N (2022). Clinical features of COVID-19 in newborns, infants, and children: a systematic review and meta-analysis. Compr Child Adolesc Nurs.

[42] Cui X, Zhao Z, Zhang T, Guo W, Guo W, Zheng J (2021). A systematic review and meta-analysis of children with coronavirus disease 2019 (COVID‐19). J Med Virol.

